# Immunohistochemical features of multifocal melanoacanthoma in the hard palate: a case report

**DOI:** 10.1186/1756-0500-6-30

**Published:** 2013-01-28

**Authors:** Luis Felipe das Chagas e Silva de Carvalho, Vitor Hugo Farina, Luiz Antonio Guimarães Cabral, Adriana Aigotti Haberbeck Brandão, Ricardo Della Coletta, Janete Dias Almeida

**Affiliations:** 1 Department of Biosciences and Oral Diagnosis, São José dos Campos Dental School, São Paulo State University (UNESP), São José dos Campos, São Paulo, Brazil; 2 Nanosciences and Advanced Materials, Federal University of ABC, Santo André, São Paulo, Brazil; 3 Department of Oral Diagnosis, Oral Pathology Division, Piracicaba Dental School, University of Campinas, Piracicaba, São Paulo, Brazil; 4 Faculdade de Odontologia de São José dos Campos – UNESP, Departamento de Biociências e Diagnóstico Bucal, Av. Francisco José Longo, 777 São Dimas, 12245-000, São José dos Campos, São Paulo, Brazil

**Keywords:** Melanoacanthoma, Mouth, Pigmented lesions

## Abstract

**Background:**

Melanoacanthoma (MA) has been described in the oral mucosa as a solitary lesion or, occasionally, as multiple lesions. MA mainly affects dark skinned patients and grows rapidly, showing a plane or slightly raised appearance and a brown to black color. The differential diagnosis includes oral nevi, amalgam tattoos, and melanomas. We report here the case of a 58-year-old black woman who presented multiple pigmented lesions on the hard palate.

**Case presentation:**

Based on the differential diagnosis of melanoma, a punch biopsy (4 mm in diameter) was performed. The material was fixed in 10% formalin, embedded in paraffin, and stained with hematoxylin-eosin or submitted to immunohistochemical analysis. Immunohistochemistry using antibodies against protein S-100, melan-A, HMB-45, MCM-2, MCM-5, Ki-67 and geminin was performed. Immunohistochemical analysis revealed strong cytoplasmic immunoreactivity of dendritic melanocytes for proteinS-100, HMB-45 and melan-A.Positive staining for proliferative markers (MCM-2, MCM-5, Ki-67) was only observed in basal and suprabasal epithelial cells, confirming the reactive etiology of the lesion. The diagnosis was oral Melanoacanthoma (MA).

**Conclusion:**

The patient has been followed up for 30 months and shows no clinical alterations. MA should be included in the differential diagnosis of pigmented lesions of the oral cavity.

## Background

In an attempt to better define the melanoepithelioma types 1 and 2 described by Bloch (1937), Mishima & Pinkus (1960) were the first to use the term melanoacanthoma (MA) [1]. According to these authors, MA corresponds to Bloch’s melanoepithelioma type 1, a rare variant of pigmented seborrheic keratosis characterized by the proliferation of melanocytes and keratinocytes in the lower layers of the epithelium [2].

The first report of oral MA was published by Schneider and coworkers (1981) [3]. Since then, MA has been described in the oral mucosa as a solitary lesion or, occasionally, as multiple lesions [2]. MA mainly affects dark skinned patients and grows rapidly, showing a plane or slightly raised appearance and a brown to black color. The differential diagnosis includes oral nevi, amalgam tattoos, and melanomas [4-8]. Histologically, MA is characterized by the proliferation of sparse melanocytes throughout the epithelium and epithelial spongiosis. An increase in the number of melanocytes in the basal layer and the presence of a chronic submucosal inflammatory infiltrate containing eosinophils are also observed [4-7]. These findings suggest the possible activation of melanocytes by an unknown mechanism that could be the link between a melanotic macule and MA and would be a reactive rather than a physiological process.

The objectives of the present study were to report a case of multifocal MA in the hard palate and to highlight the main differential diagnoses and immunohistochemical findings.

## Case presentation

A 58-year-old black woman sought the Stomatology Outpatient Clinic of the São José dos Campos Dental School in March 2008 because of a “blood stain on the roof of her mouth” (*sic*). The patient used a removable upper denture and had noted the presence of black-brownish spots on the hard palate 3 months ago. The spots had increased in size during this period and presented discrete itching when touched by the tongue.

**Figure 1:**
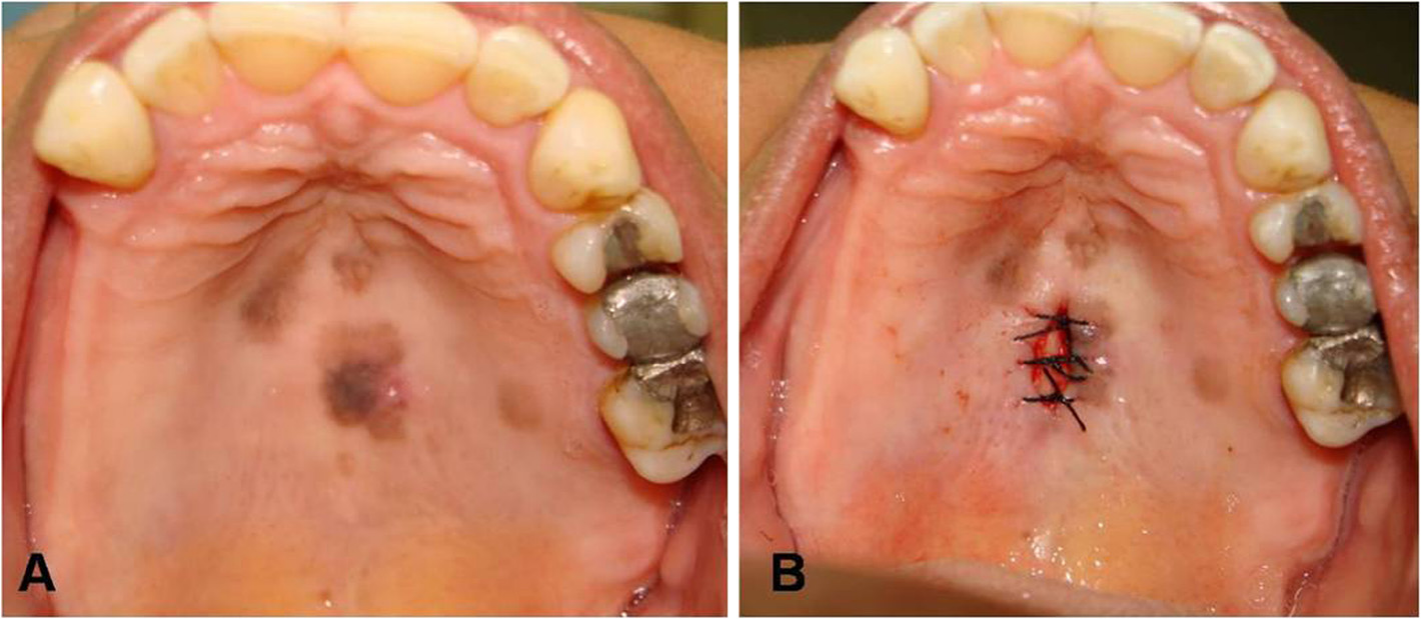
A, Photograph showing the clinical appearance of the lesion. B, Lesion afterincisional biopsy.

**Figure 2:**
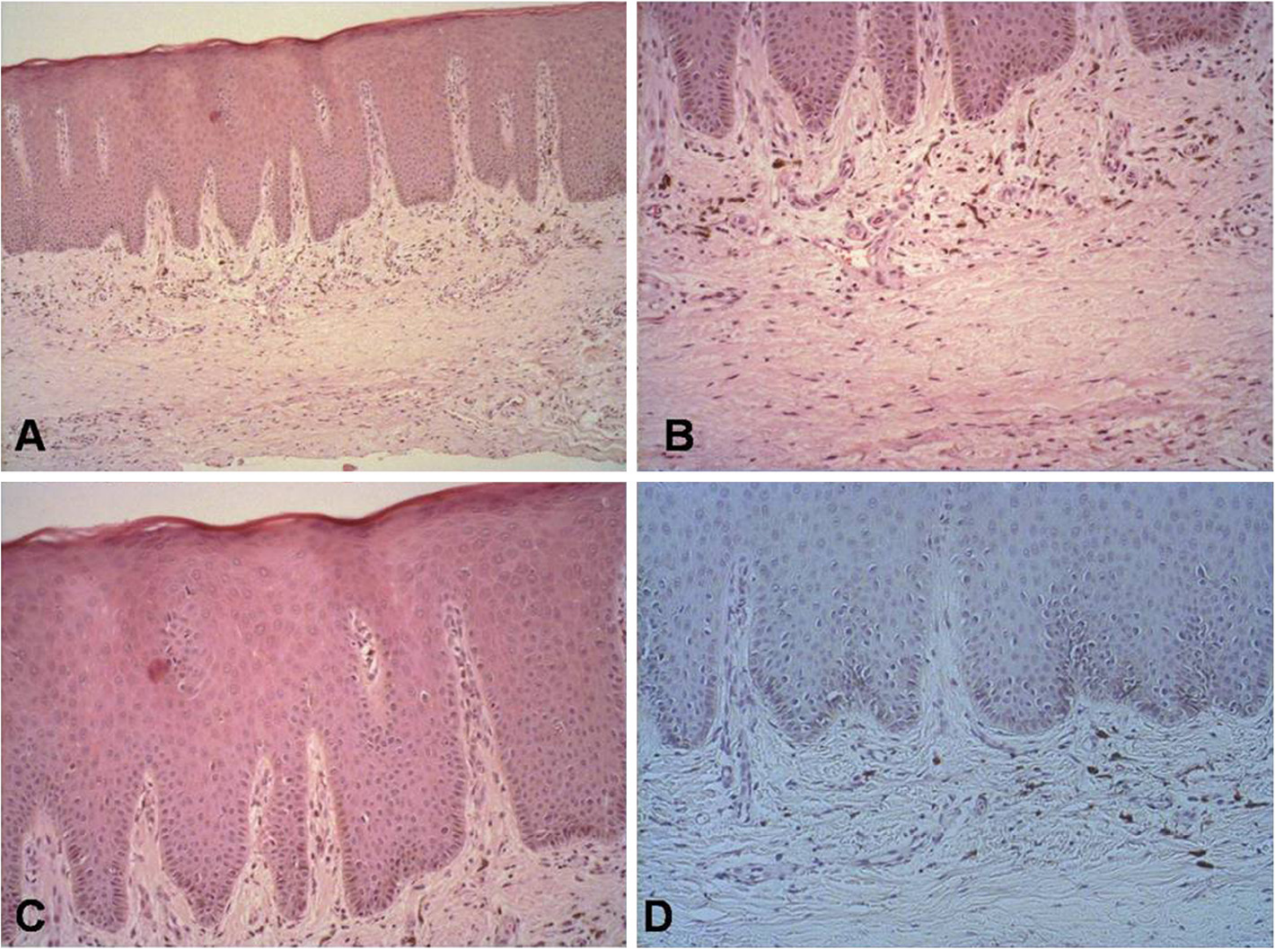
Histopathological appearance of melanoacanthoma stained with hematoxylin-eosin at different magnifications (A: 100x; B: 200x; C: 400x) and stained with periodic acid Schiff at 200x magnification (D).

Clinically, the lesions appeared as spots with imprecise borders, had a brown to dark brown color, and presented a tendency towards nodule formation (Figure 1). Radiography was non-contributory. Based on the differential diagnosis of melanoma, a punch biopsy (4 mm in diameter) was performed. The material was fixed in 10% formalin, embedded in paraffin, and stained with hematoxylin-eosin or submitted to immunohistochemical analysis [9,10]. Histopathological analysis revealed a mucosal fragment lined with hyperorthokeratinized stratified pavement epithelium. The epithelium exhibited mild acanthosis and melanin pigmentation in the basal layer. Several dendritic melanocytes were observed in the spinous layer and melanin pigment was present in the cytoplasmic processes interposed with keratinocytes. In the lamina propria consisting of fibrous connective tissue, melanophages were present in the juxtaepithelial region and a scarce and diffuse mononuclear inflammatory infiltrate was noted. In view of the histopathological findings, a diagnosis of MA was made (Figure 2).

**Figure 3:**
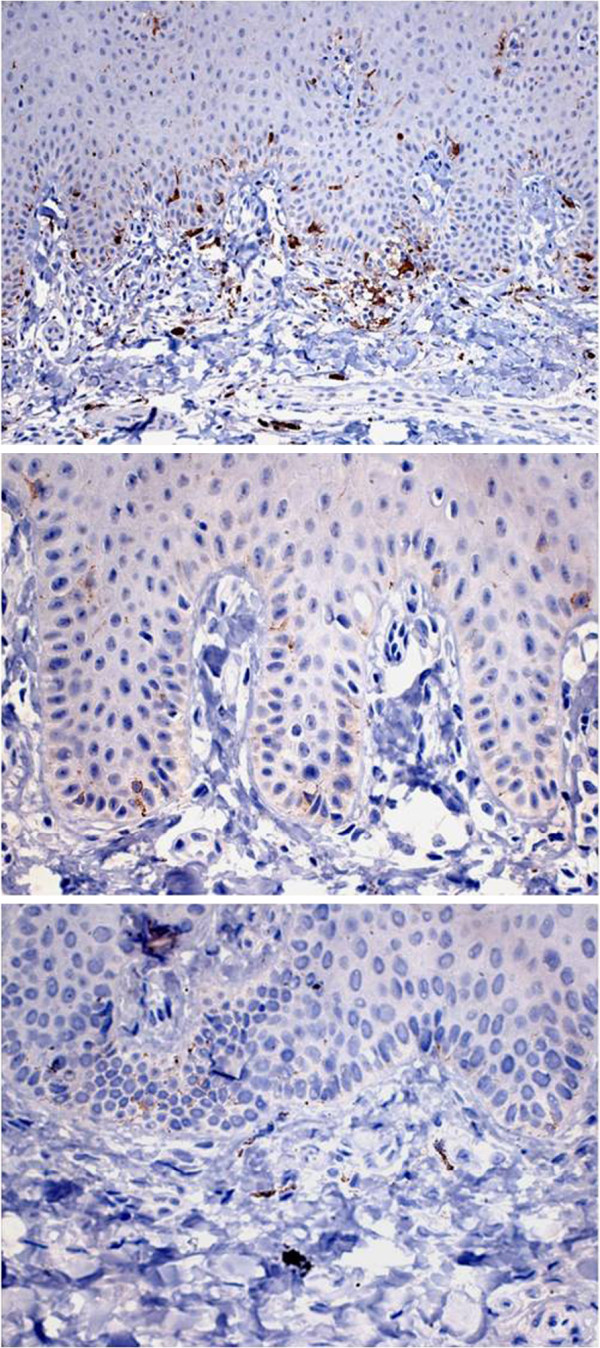
Immunostainingfor melan-A at three different magnifications.

**Figure 4:**
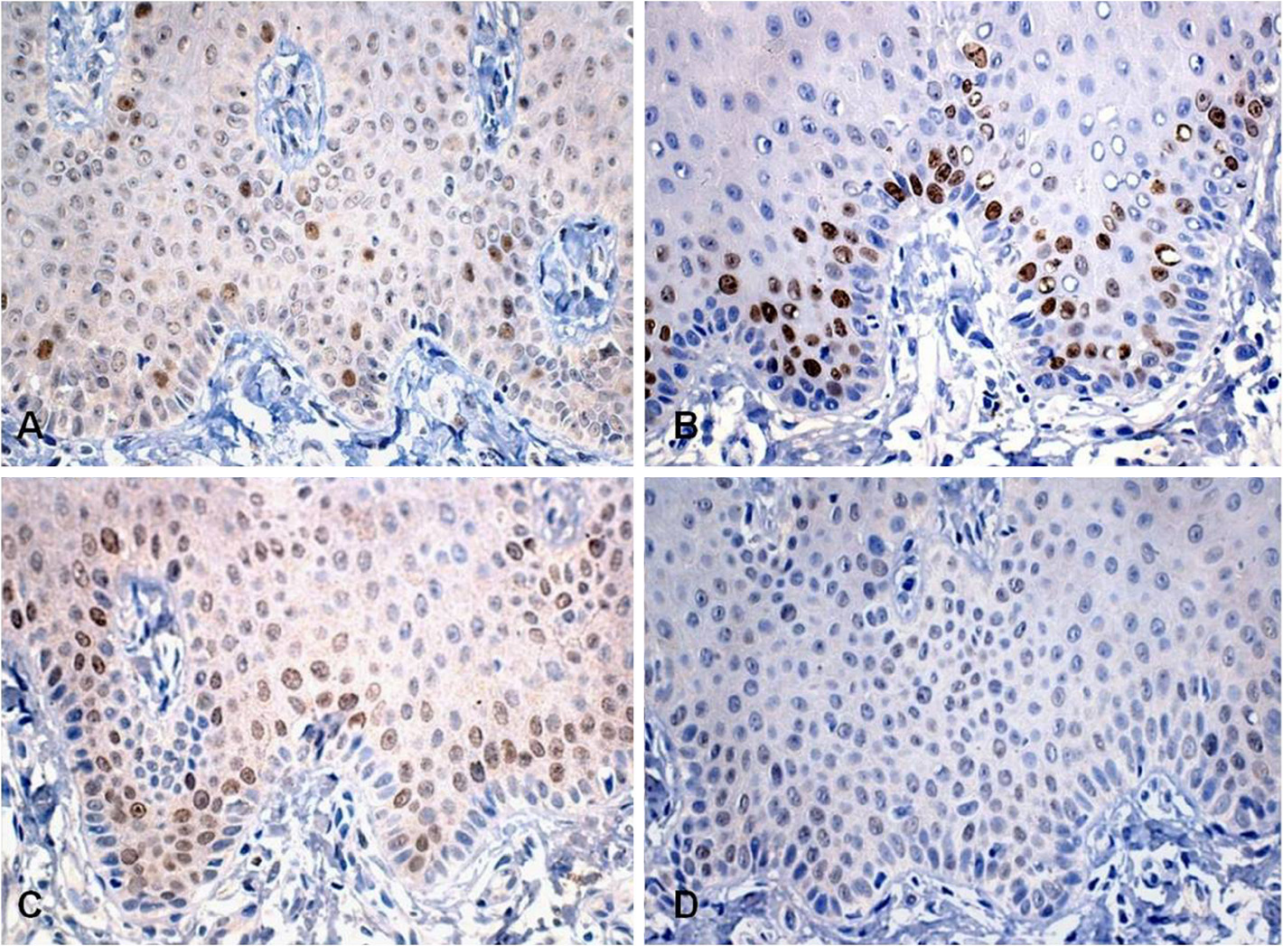
Immunostaining for geminin (A), Ki-67 (B), MCM-2 (C), and MCM-5 (D).

Immunohistochemistry using antibodies against protein S-100, melan-A, HMB-45, MCM-2, MCM-5, Ki-67 and geminin was performed for a better understanding of oral MA. Reactivity for protein S-100 was observed in Langerhans cells, melanocytes and some cells of the underlying submucosa. Immunostaining of HMB-45 and melan-A was only detected in melanocytes, with the observation of a larger number of HMB-45-positive cells. Anti-Ki-67, anti-MCM-2 and anti-geminin antibodies only reacted with cells of the basal and suprabasal layers of the epithelium, whereas MCM-5 staining was negative (Figures 3 and 4).

The biopsy region healed normally and no new intervention was necessary. The patient has been followed up for 30 months and shows no clinical alterations.

Many terms have been proposed for MA, including melanocytic reactive hyperplasia and mucosal melanotic macule, reactive type [4-7]. In a literature review, Fornatora et al. (2003) [11] analyzed 28 cases of MA and observed a mean patient age of 27.9 years (range: 9 to 54 years). Twenty-five (89.3%) of the 28 patients were black and there was a female preference (female:male ratio of 2.1:1). Although the cheek mucosa was the site most commonly affected (18 of 28 cases), MA occurred at other sites such as lip mucosa, lower lip, palate, gingiva, alveolar mucosa, and oropharynx. The size of the lesions, if reported, ranged from 0.3 to 5 cm in maximum diameter. MA presented as a smooth or slightly raised, hyperpigmented (brown to black) lesion that rapidly reached various centimeters. Traditionally, MA is asymptomatic but pain, a burning sensation and itching have been reported [4-8]. The present patient reported discrete itching upon touch.

MA is believed to be a reactive lesion that typically affects mucosal surfaces susceptible to trauma and rapidly develops after an episode of acute trauma or at a site of chronic mucosal irritation [4-8]. The rapid growth, resolution after incomplete removal, and the presence of an inflammatory infiltrate in the underlying connective tissue support the reactive nature of MA. This fact explains the higher incidence of MA in mobile mucosa vulnerable to trauma (e.g., cheek mucosa, lip mucosa, and palate). In the present case, the patient used a removable mucosa-supported upper denture. The diagnostic hypothesis was melanoma considering the clinical characteristics of the case, including rapid progression of the lesion, color, irregular contours, and tendency towards nodule formation. Although Kaposi's Sarcoma is common in hard palate it was not considered in our diagnostic hypothesis. The algorithm proposed by Kauzman et al. (2004) [12] to guide the assessment of pigmented lesions of the oral cavity on the basis of history, clinical examination and laboratory investigations includes Kaposi's Sarcoma in the group of diffuse and bilateral pigmentation with predominantly adult onset. Early lesions of Kaposi's Sarcoma appear as flat or slightly elevated brown to purple lesions and the advanced ones may appear as dark red to purple plaques or nodules that may exhibit ulceration, bleeding and necrosis [12].

Histologically, MA is characterized by the proliferation of melanocytes in the basal layer and by the presence of strongly pigmented dendritic melanocytes throughout the acanthotic epithelium. The presence of large dendritic melanocytes in the superficial portions of the epithelium is the cause of the histological resemblance with melanoma, particularly acral lentiginous melanoma. In the latter case, atypical pigmented dendritic melanocytes are irregularly distributed in the acanthotic epithelium and atypical non-dendritic melanocytes may proliferate along the basal layer (lentiginous proliferation). This type of melanoma can also exhibit a dense subepithelial lymphocytic infiltrate [5,13-15].

According to Goode et al. (1983) [13], the inflammatory infiltrate in MA exhibits eosinophilia associated with increased vascularization and mild chronic inflammation. Cases of MA usually present a slight increase of vascularization and a chronic heterogeneous inflammatory infiltrate in connective tissue. In MA, melanin is generally restricted to melanocytes, whereas adjacent keratinocytes contain no pigment. In the case of other hyperpigmented lesions such as oral melanotic macule and physiological pigmentation, melanin is transferred from dendritic epidermal melanocytes to epidermal keratinocytes that form the epidermal melanin. Once the histological diagnosis of MA is established, no further investigation is required since there are no reports of malignant transformation of MA [8].

The present histopathological findings showing no sign of malignancy agree with reports in the literature. Although some investigators emphasize the frequent occurrence of a heterogeneous inflammatory infiltrate in cases of MA [10], the present patient presented a scarce and diffuse mononuclear inflammatory infiltrate.

Immunohistochemical analysis was performed in order to better understand the etiology and behavior of MA. For this purpose, specific markers of cellular elements that might be compromised during the genesis of the disease and cell proliferation markers were used. Epithelial cells stained positive for protein S-100, demonstrating the involvement of cells of neuroectoderm origin in the etiology of MA [9]. Protein S-100 shows a sensitivity of 97 to 100% for the detection of melanoma. However, the specificity of this protein for melanocytic lesions is limited, with this marker also being expressed on neural cells, myoepithelial cells, adipocytes, chondrocytes, Langerhans cells, and in tumors arising from these cells [10]. Melan-A, a marker that recognizes normal melanocytes as well as antigens present on melanomas, was detected in the present study in some epithelial cells. Likewise, HMB-45, a melanoma marker, also stained epithelial cells but to a lesser extent than melan-A. Staining for Ki-67, MCM-2 and geminin was only detected in cells of the basal and suprabasal layers of the epithelium. Since these proteins are markers of cell proliferation, they might be responsible for the acanthotic phenomenon seen in the epithelium of MA [9]. In contrast, immunostaining for MCM-5, a marker that seems to exert a function similar to that of MCM-2, was negative.

No neoplastic progression of melanocytic lesions has been observed in the cases reported in the literature. Regression of the lesions within a period of 2 to 6 months after diagnosis has been reported after removal of the local irritating agent or after excisional and/or incisional biopsy [5]. In contrast, in the present case the lesion had not regressed and continued to be stable after 20 months of follow-up. Spontaneous resolution after elimination of the source of trauma has been reported in the literature. Therefore, investigation of local mechanical sources of irritation and their consequent elimination are recommended as the first-line treatment after diagnosis [6].

According to Carlos-Bregni et al. (2007) [5], since MA grows rapidly a biopsy is indicated to rule out the hypothesis of melanoma, among others. A biopsy is necessary for the diagnosis of any recent pigmented lesion in the oral mucosa.

## Conclusion

The immunohistochemical features of the case reported here demonstrate the importance of the application of an immunohistochemical panel to better understand the etiopathogenesis of pigmented lesions.

## Consent

Written informed consent was obtained from the patient for publication of this case report and any accompanying images. A copy of the written consent is available for review by the Editor-in-Chief of this journal.

## Competing interests

The authors declare that they have no competing interests.

## Authors’ contribution

LFCSC, VHF, LAGC and JDA examined the patient. LFCSC and VHF carried out the biopsy. JDA drafted the manuscript. RDC and AAHB participated in the design of the manuscript. AAHB performed the histological examination. RDC performed de immunohistochemistry. LACG conceived the manuscript, and participated in its design and coordination. All authors read and approved the final version of the manuscript.
